# Empathy in Medical Education: Its Nature and Nurture — a Qualitative Study of the Views of Students and Tutors

**DOI:** 10.1007/s40670-021-01430-8

**Published:** 2021-10-15

**Authors:** William F Laughey, Jane Atkinson, Alison M Craig, Laura Douglas, Megan EL Brown, Jessica L Scott, Hugh Alberti, Gabrielle M Finn

**Affiliations:** 1 Health Professions Education Unit, Hull York Medical School, York, UK; 2 School of Medical Education, Newcastle University Medical School, Tyne and Wear, Newcastle upon Tyne, UK; 3 Division of Medical Education, School of Medical Sciences, Faculty of Biology, Medicine and Health, The University of Manchester, Manchester, UK

**Keywords:** Empathy, Compassion, Communication, Medical education

## Abstract

**Context:**

Medical education is committed to teaching patient centred communication and empathy. However, quantitative research suggests empathy scores tend to decline as students progress through medical school. In qualitative terms, there is a need to better understand how students and tutors view the practice and teaching of clinical empathy and the phenomenon of empathic erosion.

**Methods:**

Working within a constructivist paradigm, researchers thematically analysed the individual interview data from a purposive sample of 13 senior students and 9 tutors.

**Results:**

The four major themes were as follows: (1) ‘the nature of empathy’, including the concept of the innate empathy that students already possess at the beginning of medical school; (2) ‘beyond the formal curriculum’ and the central importance of role modelling; (3) ‘the formal curriculum and the tick-box influence of assessments’; and (4) the ‘durability of empathy’, including ethical erosion and resilience. A garden model of empathy development is proposed — beginning with the innate seeds of empathy that students bring to medical school, the flowering of empathy is a fragile process, subject to both enablers and barriers in the formal, informal, and hidden curricula.

**Conclusion:**

This study provides insights into empathic erosion in medical school, including the problems of negative role modelling and the limitations of an assessment system that rewards ‘tick-box’ representations of empathy, rather than true acts of compassion. It also identifies factors that should enable the flowering of empathy, such as new pedagogical approaches to resilience and a role for the arts and humanities.

## Introduction

Empathy is difficult to define, but the ability to empathise is key to co-existing and co-operating with others [1]. Clinical empathy requires the physician to align to the thoughts and feelings of the patient in what has been described as a moment of ‘crossing over’ [2]. Although empathy involves the ability to ‘understand and share another person’s feelings and perspective’, there is a consensus that it must also involve a ‘self-other distinction’, whereby an empathiser does not mistake someone else’s feelings for their own [3]. There is a general consensus that empathy includes cognitive, affective, action, and moral components, though researchers disagree as to the relative contributions made by each of the components [4]. These four components act as sensitising concepts for our approach in this study [5].

Given that empathy motivates feelings of compassion and increases an individual’s drive to help [3], it follows that cultivating a sense of empathy within doctors should improve patient care. Research supports this — when patients sense empathy from their physician, they report greater satisfaction with the consultation and are likely to enjoy better health outcomes [6, 7]. Physicians too report greater satisfaction when consultations are rated as more empathic [8]

Although debate exists regarding whether empathy can be measured, there is a concerning body of research which suggests empathy declines as medical students progress through their training. Hojat et al. [9] and Newton et al. [10] report declines in mean empathy scores after the third year of medical school, whilst a recent review of studies that sought to measure empathy changes during medical school suggested that the overall trend is for empathy scores to decline [11].

Qualitative data has added depth to this picture. Tavakol et al.’s research suggests that students perceive the formal teaching of empathy to decline in the senior years of medical school [12], giving way to a curricula bias which prioritises the biomedical aspects of clinical care [13]. In addition to this, as students experience the harsh realities of working in busy healthcare settings, they come to recognise that there is minimal emotional support for frontline staff [14] and witness role models distancing themselves to cope with the hardships of the job [15–17] — learnings from what has been termed the ‘hidden curriculum of medical education’ [18].

Hafferty and Franks [18] argue that the process of socialising students into the cultural norms of medical practice — of which empathic communication is one example — is only partly driven by the curriculum that a medical school officially lays out through lectures and seminars, otherwise known as the *formal curriculum.* Instead, values are communicated to students through less structured means, particularly the setting of clinical placement. Here, teaching and learning occur mostly in the *informal curriculum*, within which students are exposed to subtle, hidden lessons which ‘can often be antithetical to the goals and content of the courses that are formally offered’ [18]. These latent lessons are part of the *hidden curriculum*, they operate at the level of stealth, and are more powerful for it [18]. In the hidden curriculum, students witness negative role-modelling and the pressure of time overriding the requirement for compassion — experiences which risk turning them away from empathy [19].

With these negative influences from the hidden curriculum and the pressures of getting through medical school, future doctors’ capacity for compassion may be fatiguing even before they qualify. Several researchers have suggested this burnout comes hand-in-hand with cynicism, a hardening of the heart, leading to ‘ethical erosion’ [20–22] — a phenomenon whereby medical students and doctors become less morally sensitive and ethically aware [23, 24]. Strategies which foster a culture of empathy within medical school are, therefore, of the utmost importance.

It is generally agreed that more *qualitative* inquiry is needed to better understand how empathy is shaped by medical education, and the reasons for empathic decline [25, 26]. Further, although research suggests that empathy can be taught [27, 28], there has been relatively little research regarding the pedagogical strategies which students and faculty perceive as acceptable and valuable.

Recent research has suggested pedagogical strategies which place emphasis on the empathic statement (stock phrases such as ‘I’m sorry to hear that’) can lead students to experience ‘empathic dissonance’, defined as ‘the mental discomfort experienced by the act of making expressions of empathy that are not sincerely felt’ [29]. These same strategies can also promote ‘fake’ empathy, and are less acceptable to students than strategies which emphasise an appropriate balance of non-verbal communication [30, 31].

Acceptance of pedagogical strategy is an important precedent to engagement [32], and so further investigation of student and tutor opinions of empathy teaching may yield valuable insight into which strategies are likely to be well-received, and which strategies will best fulfil the empathic needs of students. Further research is also necessary to discern which pedagogical approaches to countering empathic decline resonate with the needs of students and staff. As such, we asked: how do senior medical students and medical school faculty conceptualise empathy and the factors which influence it, and how do these views influence perceptions of empathic pedagogy?

## Methods

### Research Approach

We adopted a relativist ontology and constructivist epistemology, which highlight the subjectivity of reality and knowledge [33–35]. We reasoned that a relativist, qualitative approach is well suited to answering the *how* questions that characterise our inquiry, detailed above. We selected Braun and Clarke’s approach to reflexive thematic analysis to analyse our data [36, 37] given its acknowledgement of the influence of the research team in data interpretation [38].

### Setting and Participants

Data were collected at Newcastle Medical School and Hull York Medical School (HYMS), during the academic years 2018–2019 and 2019–2020, following institutional ethics board approval at both sites (6182/2018). Two centres were selected to improve transferability of results. Most researchers had clinical backgrounds (JA, AC, HA, LD, MB, BL), one was a medical student (JS), and one a non-clinical Professor of Medical Education (GF). Two authors have extensive experience and expertise in qualitative research (HA, GF). Recruitment was purposive, consisting of senior medical students (years 4 and 5) and also faculty tutors from each of the medical schools. Tutors were all qualified doctors. When recruiting students, we limited recruitment to years 4 and 5, which are the final two years of medical school in the UK. These students have more placement experience than early-stage students, and so more opportunity to observe empathy in practice. Participation was voluntary. Recruitment occurred via email, social media, and word of mouth.

### Data Collection and Analysis

Researchers (JA, AC, LD, MB, WL) conducted one-to-one, semi-structured interviews with participants. Researchers followed a question stem but were open to exploring any new lines of inquiry prompted by the interview discussion. The approach was iterative, with new questions being added as data were analysed. Interviews were mainly face-to-face, or in two cases via online video or telephone (depending on participant preference). Interviews were audio recorded and transcribed verbatim by an independent professional company. Braun and Clarke’s six steps of thematic analysis were adhered to [1] Familiarisation, [2] Generating initial codes, [3] Searching for themes, [4] Reviewing themes, [5] Defining and naming themes, and [6] Producing a report [36]. Within Step 1, to enhance data familiarity, all researchers read and re-read at least one transcript, making notes in the margins of possible codes. Two researchers from each site read and re-read all transcripts from their site, fostering familiarity with local data. Anonymised data were shared across sites, and those leading analysis at each site (JA, AC, WL) also familiarised themselves with the other site’s data set. Within step 2, all researchers formally coded at least one transcript, and all transcripts were independently coded by at least two researchers, aided by the sharing of coding documents via Google Drive. Within step 3, analysis of the pooled, coded data was conducted by multiple researchers (JA, HA, LD, MB, WL, GF) and facilitated through regular online video discussions between researchers. Similar codes were collated into early sub-themes, and sub-themes reviewed alongside one another to discern connections within the data. Within step 4, team discussions facilitated review of early proposed themes and themes were defined and named as a group (step 5). WL produced a narrative report of results which was discussed by all authors synchronously and asynchronously until a final report was agreed upon (step 6).

Regular discussions also allowed researchers to judge when theoretical sufficiency occurred — the point at which the sample size was deemed sufficient to answer the study research question [39].

### Reflexive Considerations

Our approach was inductive, but most researchers were already familiar with the empathy literature. Through reflexive conversations, we shared our own thoughts about empathy and compassion, agreeing on a broad view of these concepts, to include affective, cognitive, moral, and behavioural components. All of these formed the sensitising concepts [5] around which we conceived our interview questions and data analysis.

## Results

The demographics of the 22 participants are outlined in Table 1; notably, there was a female preponderance (17 female, 5 male). We identified four major themes and 14 sub-themes from the data, as outlined in Table 2. There were very few areas where it was possible to discern significant differences in the opinions of senior students and tutors, where present these are noted in the results.

**Table 1:** Demographics

	**Tutors**	**Students**
**Newcastle**	6 (3 female, 3 male)	6 (5 female 1 male)
**HYMS**	3 (3 female)	7 (6 female, 1 male)
**Total**	9 (6 female, 3 male)	13 (11 female, 2 male)

**Table 2:** Major themes and sub themes

**Major themes**	**The nature of empathy**	**Beyond the formal curriculum**	**The formal curriculum and the ‘tick box’ influence of assessment**	**Durability of empathy**
Sub themes	Empathy vs compassion	Professionalism	Tick-box empathy — the impact of assessment	Ethical erosion
	Innate empathy	Continuity	Faking-it	Resilience
	Head vs heart empathy	Role-modelling	Simulated teaching	
	Limits to sharing and understanding		Arts and the humanities	
			Is empathy teachable?	

### The Nature of Empathy

#### Empathy Versus Compassion

There was little agreement on the distinction between empathy and compassion. Several participants believed compassion had more of an action component to it than empathy.

…compassion takes it further because you are trying to do something that is actually a loving act.

Tutor

#### Limits to Sharing and Understanding

Participants believed empathy was about putting yourself in someone else’s shoes. It involved emotional resonance, but ultimately it was not necessary to fully share patient emotions to adequately express empathy. Students also recognised that if empathy required an authentic understanding of patient context, then expressing empathy in situations for which they had no personal frame of reference was bound to be ‘disingenuous’.

Empathy is not necessarily sharing but understanding and recognising someone’s emotions

Student

So, I think it’s a bit disingenuous to say you can always have empathy for someone in the sense of really understanding their situation.

Student

#### Innate Empathy

Participants described the notion of innate empathy. There was an awareness of a natural empathy continuum, with some individuals entering medical school with more inherent empathy.

I think it’s very evident that some people innately are able to have an extra layer of either sensitivity or, emotionally, awareness and others do lack that.

Tutor

#### Head Versus Heart Empathy

Participants described how empathy starts in the ‘heart’ as an affective emotion. As students progress through medical training, they learn to control their emotions, noted as important to wellbeing. In doing so, they learn to balance their emotions cognitively, and their empathy is balanced or modulated by their ‘head’.

Heart empathy risks burnout

Student

I think compassionate people tend to go into medicine and you’ve kind of got that quality you possess, that quality of compassion, and then it's about managing it and getting that balance right … I do think it starts in the heart

Tutor

Sincerity was held to be important. As such, becoming more cognitive was a source of conflict for participants, who felt genuine empathy should come from the heart.

I think ideally [heart] but I would recognise there are situations where that’s not possible or it’s difficult

Student

Cognitive empathy was used by some as a distancing tool to maintain professional boundaries.

…[if] it was just from the heart, it might make you make unwise decisions because there are professional boundaries and you do have to be slightly careful

Tutor

The participants understanding of empathy and compassion linked closely with their perceptions of its role with professionalism as described within the next theme.

### Beyond the Formal Curriculum

#### Professionalism

Controlling one’s emotions was linked to professionalism. Participants were aware of the professional need to display ‘appropriate’ empathy without overstepping the mark.

I think people can empathise too much to a point they’re overstepping a boundary…

Tutor

Operationalising compassion as ‘part of the job’ helped participants keep professionalism in mind.

…you’ve got to be very aware that compassion is part of our job, on a day-to-day basis

Tutor

#### Continuity

Empathy was seen as easier to give if a clinician knew a patient well: the establishment of long-standing rapport facilitated a more natural connection. Unfortunately, continuity was perceived as increasingly lacking in clinical practice.

Continuity has been eroded in every area, so you haven’t got that trust, continuity or relationship… when it does come to the moment when empathy is needed, then maybe it’s more forced because you don’t actually know them very well.

Tutor

#### Role-modelling

Participants felt positive role-modelling was the best way to learn compassion and empathy in clinical practice.

My GP… gave her a double slot, and just stopped and just listened, and kind of just sat there not really saying anything, just letting her speak, and it just kind of really resonated how much he cared, and she obviously appreciated that as well.

Student

Students also described consultations lacking in empathy and doctors speaking disparagingly about patients following a consultation. Both students and tutors reflected that these interactions could, paradoxically, strengthen the resolve to be compassionate, because students saw a kind of doctor they did not want to be. However, negative role modelling also risked leading students away from compassionate care.

You come across other people who are more senior to you that become a bit more off-hand about things, and I think we are taught a lot as medical students to copy the practice that we see, and sometimes I don’t think we are skilled enough to know what’s good practice and what’s bad practice at that moment.

Student

Tutors recognised the power of positive role modelling, but felt the pressure of time and other stresses could hinder their own efforts to be such role models.

### The Formal Curriculum and the ‘Tick Box’ Influence of Assessment

#### Tick Box Empathy

Narratives detailed the unintended consequences of assessments, such as Objective Structured Clinical Examinations (OSCEs). Assessments were deemed to lead to a reductionist, or ‘tick box’ approach to empathy.

…in an OSCE, you’re just trying to tick a box, aren’t you? And you drop in a statement ‘oh that must be really hard?’ and I think there is probably quite a lot of that. But then… everyone is under a lot of stress. 

Student

Alternatives to assessing empathy in OSCEs were suggested, including more continual forms of assessment during placements.

Do it as like a longitudinal thing, don’t… take a snapshot on a single day

Tutor

#### Faking-It

The propensity to ‘fake’ empathy was recounted by participants. Fake empathy was particularly linked to rote statements of empathy, which can lack sincerity.

I think you have to be careful not to have stock phrases that they then stick to… I've told them that they kind of need to find what feels right for them… I suppose, heartfelt.

Tutor

#### Simulated Teaching

Participants noted simulated patients (SPs) provided an opportunity for students to learn in a safe environment. However, it was acknowledged that role-play provides an artificial environment which could contribute to the problem of tick-box empathy. Not all students felt comfortable with the performance aspect of simulation.

It is a false situation, and it depends on how comfortable you are with acting in front of people. I’m certainly not that comfortable.

Student

#### The Role of Arts and Humanities

Participants highlighted the potential for integrating arts and humanities into the formal curriculum. There was perceived value in utilising stories as a mechanism by which to facilitate meaningful discussion and explore the lived experiences of others.

These discussions and understanding these different experiences of…literature… is important in itself as an education for you as a person. And I think medical school misses out on that, because they are so focused on learning outcomes and content.

Student

#### Is Empathy Teachable?

Participants questioned whether it was possible to teach empathy and compassion to students, though aspects of compassion — such as behavioural aspects — were thought to be teachable.

I think compassionate behaviour can be taught, I’m not sure compassion can…that’s what you feel. What your teaching is how to act in a compassionate manner

Tutor

Students and tutors also felt that empathy was shaped by the innate characteristics and life experiences of students, as much as by any teaching that they receive. Ultimately, it is an interaction of these aspects that shapes a student’s maturing compassion as they progress through school.

we are not teaching in a vacuum, and this is the great difficulty

Tutor

I do think it is teachable, but then equally it comes from so many things, like it comes from life experience, it comes from how you've been brought up, so, there’s so many factors I think… and how you are with people. Yeah, you can learn, but sometimes some people are good with people, and some people just aren't

Tutor

### Durability of Empathy

#### Ethical Erosion

The general view of participants was that ethical erosion does occur, though not in all students. It was often characterised as compassion fatigue, particularly by tutors. Students cited repeat exposure of difficult circumstances, such as seeing patients with terminal illness, using expressions such as becoming desensitised, blasé and offhand when faced with suffering

I think the more you’re exposed to anything the more used to it you become… the more exposed to things like death and that kind of thing and I think that can, yeah I guess that can stop us being as compassionate

Student

By contrast, tutors were more likely to link empathic erosion to high workload, long hours and the general demands of the job.

I don’t think it’s the educational system particularly, I think perhaps it’s the clinical system that they are pushed out into… if you have to see so many patients and you’ve got a 12-hour shift … I can imagine you might find it more difficult to be empathic with somebody than if you were in a system that wasn’t quite as brutal

Tutor

There was a perception that participants needed to limit their empathic engagement to protect their own emotional wellbeing and get through medical school exams.

when you go to medical school, you kind of learn how to build a wall: like, it’s professional empathy, it’s a bit like a wall actually. It’s like a separator from patients… It’s like I’m parking my empathy, for now, while I concentrate on other things.

Tutor

However, not all participants thought empathy declined with progression through medical school, with one arguing the change in empathy was more about maturation than erosion.

I don’t think there is an erosion of empathy, I think it matures into something that’s a bit more substantial and less introspective and more patient focused.

Tutor

A variety of suggestions emerged from the interviews related to how ethical erosion could be resisted, including the need for students to be aware of the phenomenon and so guard themselves against it, and the need for students to learn how to look after their own emotional wellbeing.

#### Resilience

Both tutors and students reported that when resilience was down then the reserves for empathy were also depleted.

I think we all probably have those times in our lives when we are the most stressed and working hardest. Compassion and empathy becomes something that instinctively goes.

Tutor

There was a sense of the need to balance resilience with how much empathy could be given to patients. In this sense, keeping some emotional distance from patients was seen as a protective strategy for avoiding burnout. There was seen to be a balance between the needs of patients, who require empathic investment, and the needs of the student or clinician who require some emotional distance. Losing this balance was seen to risk negative emotional transference.

I’ve had a GP partner who was very compassionate and gave patients lots of time and was always running late and eventually burnt out.

Tutor

I feel like part of burnout is to do with empathy and kind of having their problems become yours, kind of taking it home with you…

Student

Being able to switch off was seen as important for bolstering resilience, including taking breaks and holidays. Also helpful was the practice of talking problems through with colleagues, though the culture of not admitting weakness in medicine makes this difficult.

I have a tendency, if I’ve got a lot on, just to kind of close myself away and just keep on working. Whereas I know that actually just doesn’t work, and I need to just stop and take a break.

Student

There’s a lot of show and bravado in medicine, so, I don’t know that it’s… openly talked about enough.

Tutor

Students reported receiving little in the way of teaching about resilience and would welcome such an initiative, including teaching focussed on the ability to deal with the strong emotions that empathic engagement can trigger.

We’ve been taught about other aspects of emotion, for example breaking bad news and things like that, but we haven’t been taught how to deal with it emotionally in yourself if you are distressed by a situation.

Student

## Discussion

We began this research by asking how we conceptualise clinical empathy and how empathy is influenced by the teaching and practice of medicine. Our data suggest that empathy often involves finding a delicate balance between opposing concepts – for example, emotional giving and self-preservation. Given this balance can be difficult to strike, empathy is seen to be fragile, like the flower in our horticultural analogy.

Part of this question of balance is the extent to which clinical empathy should be cognitive (centred on understanding), or affective (centred on feeling). These data suggest that without a level of feeling, there is the danger that purely cognitive empathy will seem insincere, especially in the case of rote statements of empathy. This echoes the findings of other qualitative studies [30, 31] which outline the limitations of purely cognitive empathy, suggesting rote statements can be used as insincere substitutes for authentic empathic engagement.

**Fig. 1:**
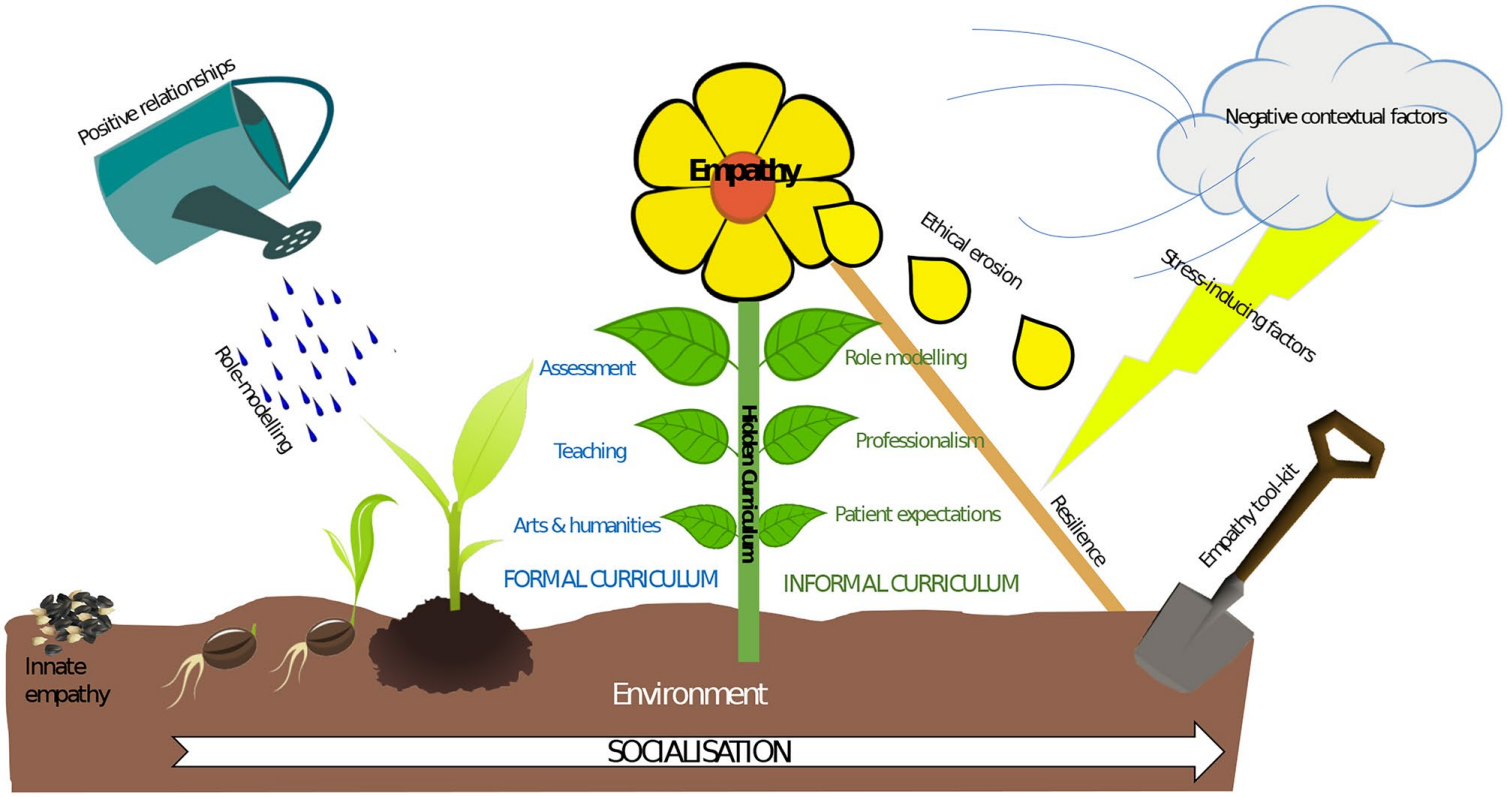
The empathy garden. Empathy is represented by a flower. All students start their developmental journey with innate empathy, demonstrated by the seeds. The growth of the seeds to a flower occurs as students navigate the formal (blue) and other than formal curricula (green) and their constituent parts (the leaves). Central to how these curricula experiences intersect is the stem, depicting the hidden curriculum. Empathy is nurtured by positive relationships and role-modelling, including positive consultations with patients, continuity, and support from peers and tutors — represented by the watering can and droplets. If provided with the right tool-kit (spade), through formal teaching, positive role-modelling, and peer and tutor support, students can become more resilient (the stake). Resilience is tested by stress-inducing factors (the lightening), such as the stress of recurring examinations and NHS pressures, including long-hours and high workload. Over time, or without support, with exposure to negative contextual factors (the wind) including negative-role modelling and the cynicism that accompanies the practice of faking empathy, there is an ethical erosion and decline in empathy — symbolised by the falling petals. The growth and erosion of empathy occurs through the process of socialisation — this includes exposure to role-models, patients, tutors, or tacit and implied experiences from the hidden curriculum

Although previously the model for the professional delivery of empathy centred on ‘detached concern’ [40], more recently Halpern [30] has suggested the idea that doctors can put their feelings entirely to one side is neither likely, nor desirable (Fig. 1). Instead, Halpern advocates emotional resonance and ‘compassionate curiosity’ for the patient’s circumstances, ideals which require a measure of affective empathy [41]. Our data suggest that a barrier to achieving this is the perception that any emotional investment in the patient’s predicament could be detrimental to the student or doctor. Both students and tutors report that they do not feel equipped to deal with the strong emotions linked to sincere empathic engagement and worry that this could leave them more susceptible to burnout. Whilst this concern makes intuitive sense, the evidence regarding affective empathy and the risk of burnout is contradictory. Whilst some research supports this link [42, 43], other evidence suggests that doctors who exhibit more affective empathy are also those who are at the least risk of burning out [44]. This is an area that would benefit from further research.

While based on only two UK medical schools, our data suggest that resilience is not taught in any systematic way. Although resilience (like empathy) is difficult to measure, there is some evidence that specific educational workshops and the techniques of cognitive behavioural therapy (CBT) can improve resilience [45]. Future studies could consider whether these interventions may allow for greater willingness to engage in affective empathy. Such research could help guide pedagogical strategies and the future integration of effective resilience teaching into medical school curricula. Currently, the perception that students do not feel equipped with strategies to deal with emotional aspects of patient contact, expressed clearly in our data, may be deterring students from being fully open to empathic consulting.

This study underlines of the key role played by the hidden curriculum: for example, positive role-modelling, accompanied by appropriate student reflections, were felt to be the most direct ways to enhance a student’s appreciation of empathy, and there is evidence in the wider literature to support this [15–17]. Tutors were aware of this, and to some extent they felt the burden of it, highlighting that stressful work environments, coupled with a lack of time, meant they could not always live up to the empathic role models they aspired to be. Therefore, whilst desirable, positive role-modelling may not always be easy to attain.

In common with previous findings [15, 46], our data also suggest that students witness frequent examples of negative role-modelling, including hearing doctors make derogatory remarks about patients after displays of seeming empathy and witnessing doctors who communicate with minimal empathy in their consultations. Negative role modelling risks giving the impression that empathy is unimportant. It is interesting, however, that some students in this study, witnessing negative role modelling, used reflection to strengthen their resolve that they themselves would never want to practice in a way that seems so lacking in empathy. Other researchers have also reported how students can take positive reflections from negative experiences [46] and educators should consider promoting the use of reflective practice to help combat the adverse influences less than compassionate role models.

Previous research has cast doubt on the assessment of empathy, especially in OSCEs [30, 31]. Students in this study have, once again, raised the issue of ‘tick box’ assessments, noting that simply making a rote statement of empathy, regardless of whether it was meant, is enough to secure the marks. The implied message within the hidden curriculum here is that a performance of empathy — and OSCEs of course occupy the level of ‘show’ in Miller’s Pyramid [47] — is all that is really needed. Assessing empathy in a way that rewards rote empathic statements may ultimately be to the detriment of the teaching of compassionate communication in medical education.

### Limitations

The principal limitation of this study is that it is restricted to two UK medical schools. Empathy has cultural aspects, and the findings may have less relevance for schools in other settings, particularly Eastern schools. For example, research on empathic decline suggests it is a Western school phenomenon — Eastern schools do not share the same decline [11]. Furthermore, there was a preponderance of female participants in this study (17 females, 5 males), which may affect the transferability of results given there is evidence to suggest that, on average, females are more empathic than males [48–50].

## Conclusion

This qualitative study outlines a number of educational factors which all have the potential to shape student empathy during their time in medical school. We have depicted empathy as a flower, emphasising its fragile nature, and its susceptibility to potential perils that reside mainly in the hidden curriculum of medical education. We have also described the perceived trade-off between empathy and resilience, that giving the former reduces the latter. This perception may or may not be correct. However, it remains significant barrier to empathic engagement and requires more research.

Based on the findings of this study, we propose a number of key points for educators (Box 1).

**Table:** 

Box 1 Key Points
• Students arrive at medical school with their innate empathy which is then prone to influence as they commence their socialisation into medicine. There is the potential for growth or decay.
• Sincere empathic communication involves a balance of cognitive and affective components, though tutors and students fear that engaging emotions risks burnout and compassion fatigue.
• Research into resilience should include strategies that embolden students to emotionally engage with patients.
• Educators should heed the importance of role modelling.
• Appropriate reflective practice may help augment the influence of positive role models and combat the adverse messages from negative role models.
• Educators should strive to reform the current assessment system which implies that shows of empathy are all that is needed. The show of compassion is not the same as the true act.
